# MRD-YOLO: A Multispectral Object Detection Algorithm for Complex Road Scenes

**DOI:** 10.3390/s24103222

**Published:** 2024-05-18

**Authors:** Chaoyue Sun, Yajun Chen, Xiaoyang Qiu, Rongzhen Li, Longxiang You

**Affiliations:** School of Electronic Information Engineering, China West Normal University, Nanchong 637009, China; sunchaoyue@stu.cwnu.edu.cn (C.S.); qiuxiaoyang@stu.cwnu.edu.cn (X.Q.); lee1225rz@stu.cwnu.edu.cn (R.L.); ylxzxx@stu.cwnu.edu.cn (L.Y.)

**Keywords:** autonomous vehicle, computer vision, object detection, multi-modality fusion

## Abstract

Object detection is one of the core technologies for autonomous driving. Current road object detection mainly relies on visible light, which is prone to missed detections and false alarms in rainy, night-time, and foggy scenes. Multispectral object detection based on the fusion of RGB and infrared images can effectively address the challenges of complex and changing road scenes, improving the detection performance of current algorithms in complex scenarios. However, previous multispectral detection algorithms suffer from issues such as poor fusion of dual-mode information, poor detection performance for multi-scale objects, and inadequate utilization of semantic information. To address these challenges and enhance the detection performance in complex road scenes, this paper proposes a novel multispectral object detection algorithm called MRD-YOLO. In MRD-YOLO, we utilize interaction-based feature extraction to effectively fuse information and introduce the BIC-Fusion module with attention guidance to fuse different modal information. We also incorporate the SAConv module to improve the model’s detection performance for multi-scale objects and utilize the AIFI structure to enhance the utilization of semantic information. Finally, we conduct experiments on two major public datasets, FLIR_Aligned and M^3^FD. The experimental results demonstrate that compared to other algorithms, the proposed algorithm achieves superior detection performance in complex road scenes.

## 1. Introduction

With the gradual maturation and widespread application of autonomous driving technology, safety has become a major concern. Object detection is one of the core technologies in autonomous driving [1]. Autonomous driving relies on detection algorithms to perceive the surrounding environment based on images captured by visual sensors and to make decisions and plans for vehicle behavior. Currently, autonomous driving mainly relies on visible light images, which perform well in bright and clear scenes but poorly in scenarios with limited visibility, such as night-time, rainy, or foggy conditions [2]. This can lead to missed or false detections of road objects, posing a threat to the safety of road users. Some researchers consider using infrared images for road object detection tasks. Infrared images are rich in semantic information, less affected by environmental interference, and perform better than visible light images in scenes with limited visibility. However, infrared images lack detailed information, resulting in inferior detection performance compared to visible light images in scenes with relatively high visibility. Infrared images are rich in object semantic information and less affected by environmental interference, while visible light images are rich in detailed information but susceptible to environmental interference. These two types of images inherently complement each other [3]. Effectively integrating and utilizing the information from both types of images, allowing each to leverage its strengths, can significantly improve the detection performance of current models in complex and dynamic road scenes [4]. Therefore, object detection based on multispectral images has become one of the current research hotspots [5,6].

In previous research, researchers have proposed various approaches to effectively utilize multispectral information, which can be broadly categorized into three types. The first type is pixel-level fusion, which focuses on fusing images at the pixel level and then inputting the fused images into the detector for detection [7]. The second type is feature-level fusion, where fusion occurs during the feature extraction process of the two modal images [8,9]. The third type is decision-level fusion, where detectors are used separately to detect the two modal images, and their detection results are fused using a certain strategy [10]. Pixel-level fusion tends to have suboptimal performance due to difficulties in perfect image registration, while decision-level fusion requires the simultaneous use of two detectors, leading to excessively large parameter sizes. Feature-level fusion has gradually become the primary focus of current researchers [11].

In the context of feature-level fusion, researchers mainly focus on two key issues: fusion timing and fusion strategy. Exploration of fusion timing has been sufficiently deep [12,13] in current research, but further research is still needed on fusion strategies. Traditional methods [14,15] employ proportional cascading, serial connection, and other methods for fusion, which are effective to some extent but have limitations in fully leveraging the advantages of multi-modal data and suffer from information redundancy and poor reliability. Some scholars have proposed illumination-aware weighted fusion methods [16], which effectively address illumination changes but still have limitations in scenarios such as foggy or rainy weather. Additionally, some researchers have proposed guiding fusion using attention mechanisms to better integrate multi-modal information [17]. While these approaches have achieved certain results, they overlook the fact that irrelevant information from both modalities is also fused during the process, which negatively impacts detection performance. Therefore, there is a need to explore more effective methods to better integrate multi-modal information. Furthermore, previous studies on multispectral detection algorithms often focus solely on improving the fusion of multi-modal information, neglecting inherent issues in detection networks for road detection, such as poor performance in detecting multi-scale objects and underutilization of semantic information.

This paper proposes a dual-modal object detection algorithm, MRD-YOLO, based on YOLOv8, to address the issues of insufficient fusion of multi-modal information and poor performance in road object detection encountered by previous multispectral detection algorithms. To tackle the problem of underutilization of multi-modal information, we design a dual-modal feature fusion module based on high-frequency information and attention guidance. To address the issues of poor detection performance on multi-scale road objects and inadequate utilization of semantic information, we replace the convolution layers and SPPF layer of the original backbone network with SAConv [18] and AIFI [19] structures. We select YOLOv8s as our baseline model. Furthermore, to validate the effectiveness of our proposed algorithm, conduct experiments on two widely used dual-modal road detection datasets, FLIR_Aligned [20] and M^3^FD [21], and compare it with current state-of-the-art dual-modal detection algorithms. The experimental results demonstrate the excellent detection performance of our proposed algorithm in complex scenarios. The main contributions of this paper can be summarized as follows:

- To address the issue of insufficient fusion and effective utilization of multi-modal information by traditional fusion methods, we propose the BIC-Fusion module based on high-frequency information and attention guidance.
- To tackle the problems of poor detection performance on multi-scale road objects and the inadequate utilization of semantic information overlooked by traditional multispectral detection algorithms, corresponding improvement measures are proposed.
- Based on the proposed fusion method and improvement strategies, we developed a novel multispectral object detection algorithm called MRD-YOLO. Experimental tests were conducted on public datasets to successfully validate the superiority of the algorithm proposed in this paper.

## 2. Related Works

### 2.1. Object Detection Models

Current object detection algorithms can be broadly categorized into two main types: Two-stage and One-stage. Two-stage algorithms typically consist of two steps: first, determining the region where the object is located, and second, classifying the objects within that region. Although this approach yields better detection accuracy, it tends to be slower. Representative examples include R-CNN [22] and Faster R-CNN [23]. On the other hand, One-stage algorithms combine object localization and classification into a single regression problem, completing both tasks in one step. These algorithms are known for their faster detection speed but may have relatively lower detection accuracy compared to Two-stage algorithms. The most representative example of One-stage algorithms is the YOLO (You Only Look Once) algorithm. Considering the real-time detection requirements of certain applications, we chose the YOLO series algorithm, which is the most representative and performs well in terms of efficiency, to complete the detection task.

In 2016, the YOLOv1 [24] algorithm was introduced, and it transformed the detection task into a regression problem, significantly improving the efficiency of object detection. Subsequently, in 2017 and 2018, YOLOv2 [25] and YOLOv3 [26] were successively released. Among them, YOLOv3 greatly enhanced the detection performance of the YOLO algorithm by using excellent backbone networks, feature pyramids, anchor-based strategies, and other outstanding techniques. This advancement enabled the YOLO algorithm to achieve excellent detection results while maintaining high real-time performance, attracting widespread attention at the time. In 2020, the YOLOv4 [27] and YOLOv5 [28] algorithms were introduced. YOLOv5 extensively adopted state-of-the-art detection strategies, including data augmentation, replacing the original PAN structure with the FPN-PAN structure and using advanced loss functions. These strategies not only ensured real-time performance but also further improved the detection effectiveness of the YOLO series algorithms. YOLOv5 became one of the most favored algorithms in the field of object detection in the following years. Subsequently, researchers continued to refine and improve the YOLO algorithm, leading to the introduction of subsequent versions such as YOLOX [29], YOLOv6 [30], YOLOv7 [31], and so on. These algorithms iteratively enhanced the detection performance of object detection algorithms in general-purpose object detection tasks.

In 2023, YOLOv8 [32] was introduced by the Ultralytics team, who previously developed YOLOv5. Compared to YOLOv5, YOLOv8 incorporates several significant improvements. In the backbone network, YOLOv8 replaces the original c3 structure with a c2f structure, enhancing the design efficiency. Additionally, in the Head layer, YOLOv8 adopts more efficient decoupled heads instead of coupled heads used in YOLOv5. It also transitions from the anchor-based concept to the anchor-free approach and replaces the original BCE_Loss in the loss function with DFL and CIOU as regression losses. These enhancements result in YOLOv8 achieving higher accuracy and faster speed in object detection. Notably, YOLOv8 surpasses YOLOv7 on the classic CCOO public dataset, establishing itself as one of the most advanced object detection algorithms to date. Furthermore, YOLOv8 introduces five versions (n, s, m, l, x) tailored for different detection tasks in various scenarios, with model parameters gradually increasing from n (the smallest) to x (the largest), corresponding to variations in detection performance, with n offering the lowest performance and x offering the highest.

However, the aforementioned detection algorithms are limited to detecting single-modal data. This reliance on single-modal detection inevitably faces constraints inherent to each modality. For instance, RGB-based object detection struggles in adverse conditions such as rain, fog, and night-time, where environmental factors and lighting severely impact imaging quality, resulting in poor detection performance. On the other hand, infrared-based object detection, while relatively stronger in limited visibility scenarios compared to RGB images, lacks detailed information and performs poorly in typical scenes compared to visible light. Therefore, single-modal object detection algorithms struggle to effectively address the complexities and variations in road scenes.

### 2.2. Multispectral Object Detection

To address the issue of poor detection performance in complex scenarios due to the limitations of individual modalities in single-modal detection, multispectral object detection has emerged. Multispectral object detection algorithms integrate both RGB and infrared modalities, utilizing the salient information from infrared images to mitigate the negative impact of environmental conditions on detection while leveraging the detailed information from RGB images to further enhance performance in various complex scenarios. The core of multispectral detection lies in effectively fusing different modalities of information, which can be categorized into pixel-level fusion, feature-level fusion, and decision-level fusion based on fusion timing [33]. Pixel-level fusion requires high registration accuracy between different modal images, limiting its effectiveness in improving detection performance, especially in real-time scenarios [34]. Decision-level fusion involves using two separate algorithm networks and then combining the results, resulting in excessive parameters and impracticality in real-time applications. Therefore, feature-level fusion has become the primary focus of current researchers. Wagner et al. [13] initially proposed early fusion and late fusion frameworks. Afterwards, Hong et al. [35] discussed three fusion timings: early, middle, and late fusion, concluding that middle and late fusion methods are more effective. Subsequent researchers have mostly adopted middle fusion methods. In addition to studying fusion timing, researchers have also focused on fusion methods. In the early stages, some researchers [14,15] adopted relatively simple fusion methods such as proportional cascading, element-wise addition, and element-wise multiplication, which yielded initial results in some scenarios, demonstrating the effectiveness of multi-modal information in enhancing detection performance to some extent. However, these simple fusion methods have a limited utilization of both modalities. Zheng et al. [36] proposed GFD-SSD, a multispectral detection algorithm based on the SSD algorithm, using two different gating fusion units to learn the detection effects of different modalities in different scenarios. Zhang et al. [17] introduced GAFF, a method based on attention-guided feature fusion. Yun et al. [37] proposed Infusion-Net based on high-frequency information acquisition and YOLOv7 algorithm. Xie et al. [38] designed a multispectral detection algorithm, YOLO-MS, based on the YOLOv5 framework and attention-guided fusion.

However, most of the above methods overlook the fact that features irrelevant to the target are also fused during fusion, leading to negative effects on the detection performance after fusion, indicating that fusion strategies still need improvement. Additionally, the aforementioned researchers only focused on fusion itself, simply embedding the fusion module into the established object detection algorithm, overlooking issues inherent in the detection algorithm itself, such as poor performance in detecting multi-scale targets and insufficient utilization of semantic information in road detection tasks. To address these issues and enhance the detection performance in complex road scenarios, this paper proposes and designs the MRD-YOLO algorithm. The algorithm utilizes the BIC-fusion module to interactively fuse effective feature information and employs attention mechanisms to guide the fusion of multi-modal information, enabling the full utilization of multi-modal information. Furthermore, corresponding improvements are proposed to address the inherent issues of the YOLOv8 algorithm, such as poor performance in detecting multi-scale targets and insufficient utilization of semantic information. Compared to other multispectral algorithms, MRD-YOLO demonstrates superior detection performance in road detection tasks in complex scenarios.

## 3. Methods

**Figure 1:**
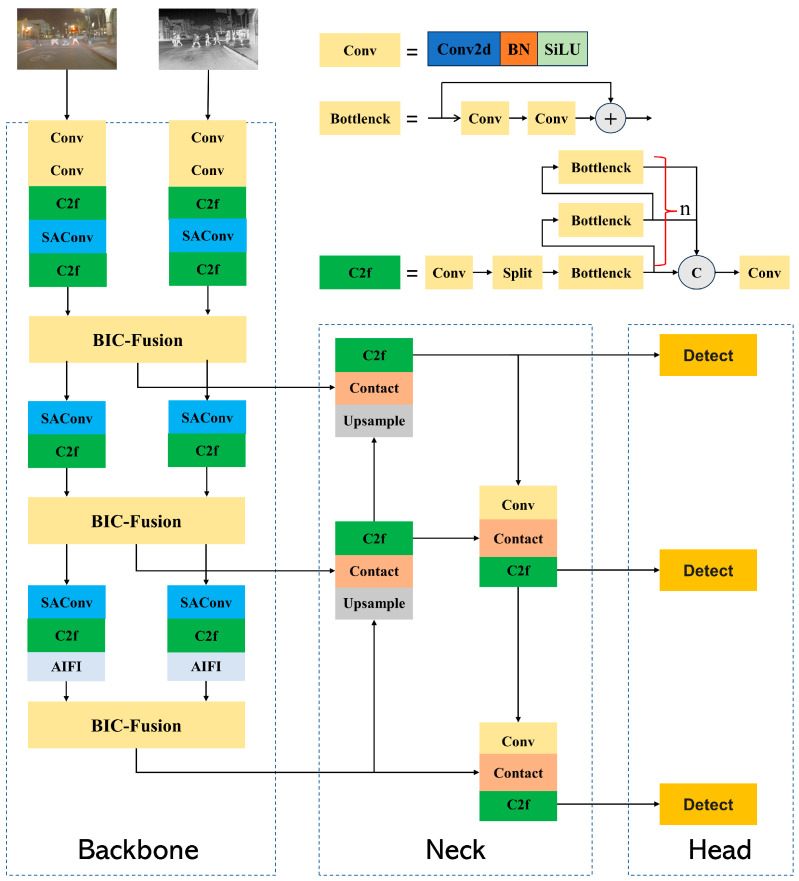
Overall structure of MRD-YOLO.

This section will provide a detailed explanation of the network architecture and its key components of MRD-YOLO. MRD-YOLO is designed based on the YOLOv8 network architecture, consisting of three main parts: backbone, neck, and head. The overall structure is illustrated in Figure 1.

In the backbone part, we have modified the original single-modal backbone used for processing single-modal data into a dual-backbone structure to extract both RGB and infrared (IR) dual-modal information. We introduce the BIC-Fusion fusion module to facilitate information interaction and feature fusion between the dual-modal information, effectively filtering out redundant information. This approach maximizes the utilization of dual-modal information, thereby enhancing the detection performance of the algorithm in complex scenarios. Additionally, to address the issues of poor detection performance for multi-scale targets and underutilization of semantic information inherent in the detection algorithm, we replace some Conv structures in the backbone network with SAConv. This strengthens the algorithm’s ability to extract features for multi-scale targets. Furthermore, we replace the SPPF structure with the AIFI structure to enhance the utilization of semantic information in deep feature maps, effectively improving the detection performance of the algorithm for scale-variable road targets. It s important to note that in the last BIC-Fusion of the Backbone part, we only output the final fused feature map without separately outputting RGB and IR branches. In the neck part, we employ the FPN-PAN structure to perform multi-scale fusion of the fused dual-modal features at three different scales, enhancing the detection performance for targets of various scales. In the head part, we utilize the original detection head of the YOLOv8 network to predict target positions, categories, and other information. Next, we provide detailed explanations of the BIC-Fusion module, SAConv, and AIFI, which are the main modules used in the network.

### 3.1. BIC-Fusion Module

**Figure 2:**
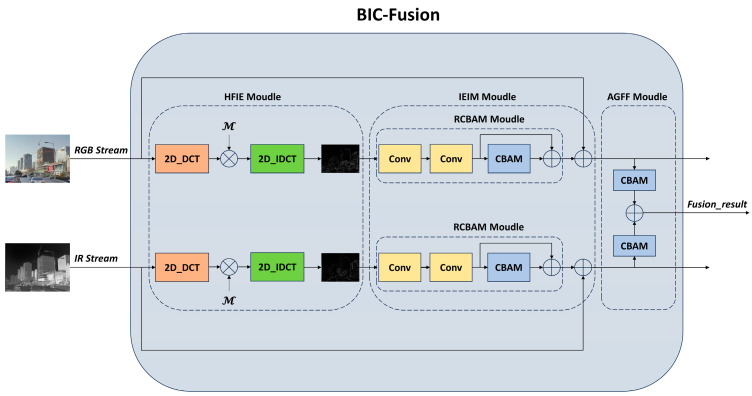
Structure of BIC-Fusion.

To better integrate the information from two modalities of images, we designed the BIC-fusion module in this paper. The module consists of three parts: the HFIE module, the IEIM module, and the AGFF module. The HFIE module is responsible for extracting high-frequency effective information, the IEIM module enhances and interacts with features, and the AGFF module outputs the final fused feature map. The overall structure is illustrated in Figure 2.

HFIE Module (High-Frequency Information Extraction Module): High-frequency information in images refers to areas where the grayscale values change dramatically in 2D images. Compared to low-frequency information, high-frequency information contains more edge and texture details, such as the contour information of targets in infrared images and the contour and detail information of targets in RGB images. These pieces of information are crucial for detection, as high-frequency information contains a significant amount of valuable data. By interacting the high-frequency information of each modality, we can reduce the negative impact of irrelevant information fusion on detection, thus better leveraging the advantages of each modality in dual-mode images. From this perspective, this paper designs an information purification module to help extract high-frequency information from both modalities. Specifically, by first using two-dimensional discrete cosine transformation (2D_DCT) to process the input feature map to obtain its frequency-domain representation, the low-frequency information in the frequency-domain image is concentrated in the upper-left corner after 2D_DCT conversion. To remove low-frequency information and retain high-frequency information, we chose to use a mask *G* to process the frequency-domain image to discard the low-frequency information located in the upper-left part of the frequency-domain image. The amount of information discarded is determined by the parameter *α*, and the size of the *α* parameter affects the amount of discarded information. If *α* is too large, valuable information may be discarded, while if *α* is too small, it may not effectively filter out useless information. Finally, through inverse transformation (2D_IDCT), the frequency-domain image is converted back to the feature map, resulting in a new feature map rich in high-frequency useful information. The specific formulas for the mask *G*, 2D_DCT, and 2D_IDCT are as follows:

(1) $$G(u,v)=\left\{\begin{array}{cc} 0, & v<-u+2\alpha w\\ 1, & \;\mathrm{otherwise}\; \end{array}\right.$$

(2) $$\begin{array}{c} 2D_{-}DCT(u,v)=\frac{2}{\sqrt{MN}}c(u)c(v)\sum_{i=0}^{N-1}\sum_{j=0}^{M-1}f(i,j)cos\left(\frac{(2i\;+\;1)u\pi }{2M}\right)cos\left(\frac{(2j\;+\;1)v\pi }{2N}\right) \end{array}$$

(3) $$\begin{array}{c} 2D_{-}IDCT(i,j)=\frac{2}{\sqrt{MN}}\sum_{u=0}^{N-1}\sum_{v=0}^{M-1}c(u)c(v)F(u,v)cos\left(\frac{\left(2u\;+\;1\right)i\pi }{2M}\right)cos\left(\frac{\left(2v\;+\;1\right)j\pi }{2N}\right) \end{array}$$

(4) $$c(u)=\left\{\begin{array}{ll} 1/\sqrt{2} & u=0\\ 1 & \mathrm{otherwise}\; \end{array}\right.$$

where Equation (1) represents the mask G, with w in Equation (1) denoting the width of the input feature map. Equations (2) and (3) represent 2D_DCT and 2D_IDCT, respectively, with F(u,v) denoting the frequency obtained after 2D_DCT conversion, and f(i,j) representing the pixel value of the pixel located at (i,j). Equation (4) is the orthogonal normalization coefficient.

**Figure 3:**
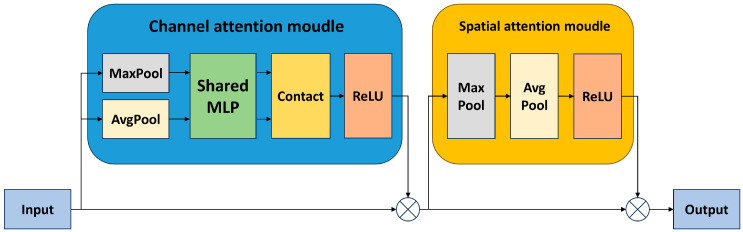
Structure of CBAM.

IEIM Module (Information Enhancement and Interaction Module): Isolated branches hinder the network from learning the correlation information between modalities. Strengthening the exchange of information between branches is crucial for learning effective complementary features. Therefore, this paper specifically designs an information interaction module for interacting effective information. This module consists of RCBAM and Add structures. CBAM [39] combines channel attention with spatial attention, allowing the network to focus on the target and its surrounding information more effectively. The specific structure of CBAM is shown in Figure 3. We combine it with the residual structure to form the RCBAM module, as shown in Figure 2. RCBAM not only enhances the network’s attention to effective features but also deepens the model to improve its learning performance. Finally, through the Add structure, the enhanced high-frequency information feature map is fused with the original feature map of the other modality, allowing the effective information of each modality to be fully interactively learned.

AGFF Module (Attention-Guided Feature Fusion Module): The AGFF Module consists of the ADDCB module, which is responsible for integrating the fused dual-modal feature maps for subsequent detection. Firstly, CBAM is applied to guide attention allocation for the two input feature maps, enabling the model to better focus on the target regions of each modality. After attention allocation, the final fusion is achieved through addition.

### 3.2. Replace Conv with SAConv

**Figure 4:**
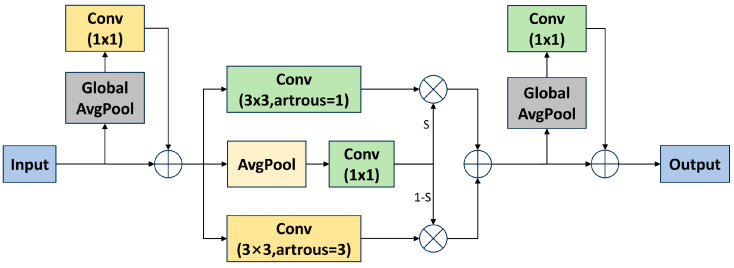
Structure of SAConv.

Dilated convolution, by introducing additional spacing (i.e., dilation) in the convolutional kernel, can enlarge the receptive field without increasing the number of parameters or computational cost. SAConv, on the other hand, improves feature extraction at different scales by applying dilated convolutions with different dilation rates. Additionally, it incorporates a switch function to control the fusion weights of these different convolutional results. Through SAConv, the network can flexibly extract features at various scales, aiding in more accurate object recognition. The overall structure is illustrated in Figure 4.

To address the issue of varying object scales in road scenes, we replaced some of the original convolutional layers in the backbone network with SAConv. This replacement effectively enhanced the model’s capability to extract features from road objects with diverse scales, thereby improving the overall performance of the model in road scenarios.

### 3.3. Replace SPPF with AIFI

**Figure 5:**
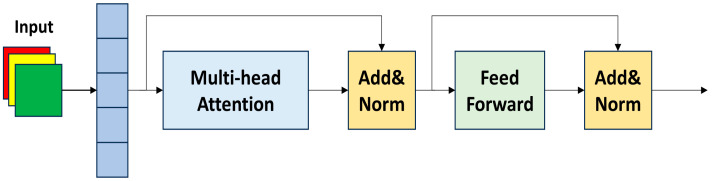
Structure of AIFI.

AIFI applies a self-attention mechanism to high-level features with rich semantic concepts, coordinating internal scale interactions among these features. This allows the network to better capture the relationships between conceptual entities in the image, thereby assisting subsequent modules in more effectively detecting and recognizing objects in the image. The overall structure of AIFI is illustrated in Figure 5.

First, AIFI transforms the input 2D image into a 1D vector for subsequent processing. Then, it employs multi-head attention to process the input, allowing the model to perform multiple sets of self-attention on the input sequence, helping the model better capture information from different positions in the sequence and improving its ability to handle long-range dependencies. Next, the processed sequence is passed through a feed-forward network (FFN) after residual connection and normalization with the serialized original input. The FFN introduces non-linear learning, enabling the network to learn complex relationships between different elements in the input sequence. Finally, the 1D vector is transformed back into a 2D form for subsequent network processing. The overall mathematical process is illustrated in Equations (5) and (6).

(5) Q=K=V=Flatten (Input)

(6) Output=Reshape (FFN(MultiHead (Q,K,V)))

By leveraging the multi-head attention mechanism and FFN structure, AIFI can facilitate intra-scale interactions among high-level features, enabling the network to better capture relationships between conceptual entities in the image. This enhances the model’s ability to handle complex semantic features, thereby improving the network’s detection performance.

## 4. Experiments

### 4.1. Dataset

The FLIR dataset [40] is a freely available thermal sensing dataset released by Teledyne FLIR to support research in autonomous driving. The dataset comprises 14,000 annotated pairs of infrared images and corresponding RGB images. All images were recorded using regular cameras and thermal imaging cameras installed on vehicles, capturing both daytime and night-time scenes. The target categories include people, cars, bicycles, dogs, etc. However, some image pairs in the original FLIR dataset are not aligned, making them unsuitable for use in bimodal detection tasks. To adapt the dataset for bimodal tasks, Zhang et al. [20] removed the unaligned image pairs from the original FLIR dataset and retained only the three common categories: people, bicycles, and cars. The processed dataset, known as FLIR_Aligned, contains a total of 5142 well-aligned pairs of visible light and infrared images, with 4129 pairs for training and 1013 pairs for evaluation.

The M^3^FD dataset, released by Liu et al. [21] in 2022, consists of 4200 pairs of aligned and annotated visible light and infrared images. It includes a total of six object categories: “person”, “car”, “bus”, “motorcycle”, “truck”, and “traffic light”. The images were captured by optical and infrared cameras mounted on vehicles, covering various challenging scenarios such as daytime, overcast, night-time, and foggy conditions. Since there is no official split for training and validation sets, we followed the split provided by [41]. The training set contained 3360 image pairs, while the validation set contained 840 image pairs.

### 4.2. Implementation Details

The experimental setup in this paper used Windows 11 as the operating system, an NVIDIA RTX 4090 GPU device, and the experimental code was built using PyTorch. The initial learning rate for training was set to 0.01, with a weight decay of 0.001. The batch size was set to 10, and the number of epochs for training on the FLIR_Aligned dataset was 150, while for the M^3^FD dataset, it was set to 400. The input image size was 640 × 640 pixels.

### 4.3. Evaluation Metrics

The experiment selected the comprehensive indicator mAP as the evaluation metric, which represents the average precision. A higher mAP indicates stronger model detection performance. The specific formula for the evaluation metric is as follows:

(7) $$P=\frac{TP}{TP+FP}$$

(8) $$R=\frac{TP}{TP+FN}$$

(9) $$mAP=\int_{0}^{1}P(R)dR$$

In the formula, TP represents the number of correctly predicted samples, FP represents the number of incorrectly predicted samples that are predicted as correct, and FN represents the number of missed detections. mAP can be divided into mAP50 and mAP50:95 based on IoU threshold criteria. mAP50 represents the mAP when the IoU threshold is 0.5, while mAP50:95 represents the average mAP over IoU thresholds ranging from 0.5 to 0.95 with a step size of 0.05.

### 4.4. Exploration Experiment of Hyperparameter α

The BIC-Fusion module relies on the parameter α of the mask G to determine how much irrelevant information is excluded. Its value determines whether we can reasonably retain effective high-frequency information and remove irrelevant information. The range of α is between 0 and 0.5. To determine a more appropriate α to ensure the effective extraction of high-frequency information, we conducted detailed experimental tests on α within the range of 0 to 0.5 using the FLIR_Aligned dataset. The experimental results are shown in Table 1.

The experimental results indicate that the model achieves optimal performance when the value of α is set to 0.3. When the value of α exceeds 0.3, the performance of the model begins to decline significantly. This is because too much information is excluded, leading to the removal of essential information along with irrelevant information, resulting in a substantial drop in detection performance. Therefore, to effectively filter out irrelevant information and retain useful high-frequency information, we set the parameter α to 0.3 based on the experimental results.

### 4.5. Ablation Study

To validate the effectiveness of the proposed algorithm and its various improvement modules, dissociation experiments were conducted on the FLIR_Aligned dataset. The results are shown in Table 2.

From Table 2, it can be observed that the model’s performance is poorest when relying solely on the RGB modality, with an mAP50 of only 61.6%. When using only the infrared modality for detection, the mAP50 improves to 74.2%. Incorporating the proposed BIC-Fusion module for multi-modal fusion leads to further improvement, with the algorithm’s performance surpassing all single-modal approaches and reaching an mAP50 of 75.7%. With the addition of AIFI, the mAP50 increases to 75.9%. Furthermore, the performance of the model improves to its best after adding SAConv, with the mAP50 reaching 76.5%. Compared to the baseline model, the algorithm proposed in this paper demonstrates significant improvements, with an increase of 14.9% in mAP50 for visible light and 2.3% for infrared images. These experimental results effectively demonstrate the effectiveness of the proposed improvement methods.

### 4.6. Comparison of Different Detectors

To validate the superiority of our proposed algorithm, we conducted experiments on the FLIR_Aligned and M3FD datasets, two widely used datasets for dual-mode road object detection. The performance of our algorithm, MRD-YOLO, was compared with that of state-of-the-art dual-mode algorithms, such as SuperYOLO [42] and YOLO-MS [38]. The experimental results presented in Table 3 and Table 4 demonstrate that even when compared with these advanced dual-mode detection algorithms, MRD-YOLO exhibits superior performance. These results strongly affirm the superiority of the MRD-YOLO algorithm.

**Figure 6:**
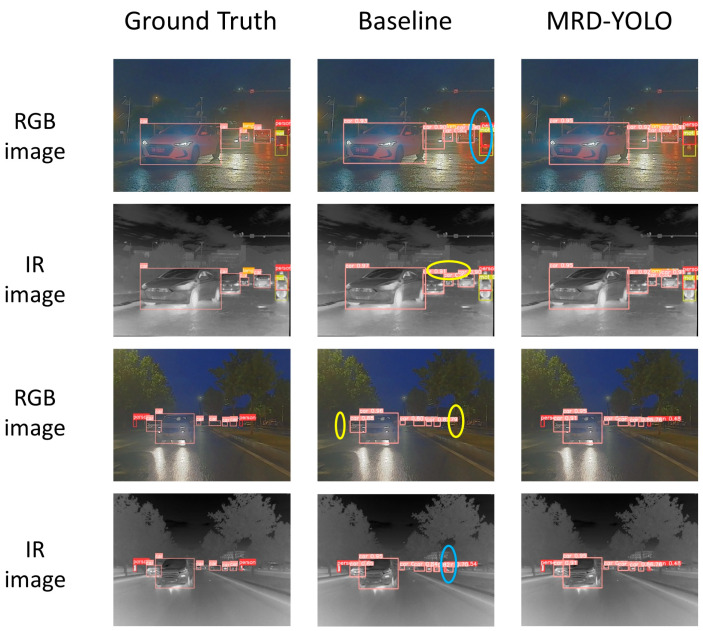
Visual comparison of detection results.

To visually demonstrate the improvement in detection performance delivered by the proposed algorithm, we present a comparison of the original model and the proposed algorithm’s detection results in both modalities in Figure 6. The left panel shows the Ground Truth, the middle panel shows the baseline model, and the right panel shows the proposed algorithm. Missed targets are annotated with yellow ellipses, while false detections are annotated with blue ellipses. From the figure, it can be observed that in scenes with complex lighting variations, the original algorithm relying on a single modality missed detections and false detections in both modalities. In contrast, the proposed algorithm, which integrates the advantages of both modalities, effectively avoids missed detections and false alarms on road targets, demonstrating superior detection performance. Additionally, the targets detected by the proposed algorithm often have higher confidence scores. The visualized results intuitively illustrate the improved detection performance of the proposed algorithm in complex road scenes.

## 5. Conclusions

This paper addresses the issue of the poor performance of current detection algorithms in detecting road objects in complex scenarios by proposing an improved dual-mode detection algorithm based on YOLOv8s, named MRD-YOLO. To address the problem of poor fusion effects in traditional dual-mode algorithms, a fusion module named BIC-Fusion is proposed to extract and fuse interactive dual-mode information. Additionally, to improve the detection performance of multi-scale objects, the SAConv module is introduced to help the model better extract multi-scale feature information. Furthermore, to address the insufficient utilization of semantic information in traditional algorithms, the AIFI module replaces SPPF to better assist the model in utilizing semantic information, effectively improving the detection performance of road objects with varying scales. Finally, the proposed algorithm is tested on the FLIR_Aligned and M^3^FD datasets for dual-mode road detection and is compared with current state-of-the-art dual-mode algorithms. The results demonstrate that the proposed algorithm exhibits excellent performance in road detection tasks in complex scenarios, effectively addressing the issue of poor performance of current models in complex road scenarios. However, the MRD-YOLO model proposed in this paper only focuses on the fusion of infrared and visible light modalities. Future work will explore the integration of more modal information to further enhance its detection performance in complex road scenarios.

## Figures and Tables

**Table 1:** Exploration experiments of hyperparameter α on FLIR_Aligned dataset.

Hyperparameter *α*	mAP50	mAP50:95
0	75.1	40.5
0.1	75.0	40.2
0.2	75.6	40.4
**0.3**	**75.7**	**40.6**
0.4	74.3	39.9
0.5	73.7	39.6

**Table 2:** Ablation experiments on FLIR_Aligned dataset. The best-performing methods are highlighted in bold.

Method	Data	mAP50	mAP50:95
YOLOv8	RGB	61.6	28.8
YOLOv8	IR	74.2	39.2
YOLOv8+BIC-Fusion	RGB + IR	75.7	40.6
YOLOv8+BIC-Fusion+AIFI	RGB + IR	75.9	40.7
**YOLOv8+BIC-Fusion+AIFI+SAConv**	RGB + IR	**76.5**	**40.9**

**Table 3:** Comparison of results on the FLIR_Aligned dataset with different algorithms. The best result is indicated in bold.

Method	Data	map@0.5	map@0.5:0.95
IV-CRN [43]	RGB + IR	72.3	-
CFR_3 [20]	RGB + IR	72.4	-
GAFF [17]	RGB + IR	72.9	37.5
CAPTM [44]	RGB + IR	73.2	-
SuperYOLO [42]	RGB + IR	74.6	39.4
YOLO-MS [38]	RGB + IR	75.2	38.3
**MRD-YOLO (Ours)**	RGB + IR	**76.5**	**40.9**

**Table 4:** Comparison of results on the M^3^FD dataset with different algorithms. The best result is indicated in bold.

Method	Data	map@0.5	map@0.5:0.95
SLBAF [45]	RGB + IR	78.9	44.3
EAEF [41]	RGB + IR	80.1	-
DAMSDet [46]	RGB + IR	80.2	52.9
RGB-X [47]	RGB + IR	81.5	-
YOLO-MS [42]	RGB + IR	85.7	55.2
**MRD-YOLO (Ours)**	RGB + IR	**86.6**	**59.3**

## Data Availability

Data are contained within the article.
